# Tablet computers to support outpatient pulmonary rehabilitation in patients with COPD

**DOI:** 10.3402/ecrj.v3.31016

**Published:** 2016-05-24

**Authors:** Thomas J. Ringbaek, Marie Lavesen, Peter Lange

**Affiliations:** 1 Respiratory Department, Hvidovre Hospital, Copenhagen, Denmark; 2 Respiratory Department, Nordsjællands Hospital, Hilleroed, Denmark

**Keywords:** COPD, telemonitoring, pulmonary rehabilitation, quality of life, technology, outcome

## Abstract

**Background:**

A minicomputer (tablet) with instructions and a training diary has the potential of facilitating adherence to pulmonary rehabilitation (PR).

**Objective:**

To evaluate the effect of adding a tablet to a classic outpatient PR programme for COPD patients.

**Methods:**

A total of 115 patients participated in a 7- to 10-week outpatient PR programme in groups of 10–12 individuals. Half of the groups were assigned to PR plus a tablet (tablet group) and the other groups were assigned to PR only (controls). Primary effect parameters were endurance shuttle walk time (ESWT) and disease-specific health status (COPD Assessment Test=CAT).

**Results:**

The change in ESWT was significantly better in the control group (mean 167 sec) compared with the tablet group (mean 51 sec) (*p*<0.01), whereas the change in CAT score did not differ significantly between the two groups (−0.6 vs. −2.3) (*p=*0.17).

**Conclusions:**

Compared with usual PR, no significant improvements were seen in the group equipped with the tablet after 7–10 weeks of rehabilitation. Future studies should focus on long-term effects.

Pulmonary rehabilitation (PR) improves physical function and quality of life in patients with chronic obstructive pulmonary disease (COPD) (1). The majority of outpatient rehabilitation programmes consist of supervised training twice a week and recommend additional unsupervised training at home at least once a week (2). Unfortunately, adherence with both home training and supervised training is often poor (3, 4).

Besides exercise training, PR aims to optimise treatment and behaviour, and includes targeted disease-specific education and counselling. The latter contains oral and written instructions (5). It is a general impression that many patients do not understand all the messages given during the rehabilitation programme or forget them already while participating and after the programme has been completed.

Mobile technologies are increasingly being used in health care for portable communication, monitoring, and education, and to facilitate adherence to chronic disease management (6). Studies of patients with asthma have demonstrated significant effect on adherence and mixed effect on clinical outcomes (6). A pilot study including 17 patients with COPD showed that it was feasible to deliver a cell phone–based exercise maintenance intervention following PR and that functional outcomes such as physical activity were maintained for 6 months after PR (7). It is suggested that telemonitoring could encourage the patients to become involved in the management of their own disease, yet this has not yet been clearly demonstrated for COPD (8).

In this study, we want to evaluate the effect of adding a tablet (minicomputer with touch screen) with instructions and training diary to a classic outpatient PR programme. The hypothesis was that, by providing a common platform for cooperation between the patient and the health professionals, a tablet could empower the patient to become an active participant evaluating his or her own improvements. In addition, the tablet could be used as an electronic diary where data are presented graphically and automatically sent to rehabilitation staff. Moreover, the device can display videos with instruction on training exercises, breathing techniques, and relaxation exercises.

## Material and methods

### Selection of patients

**Fig. 1:**
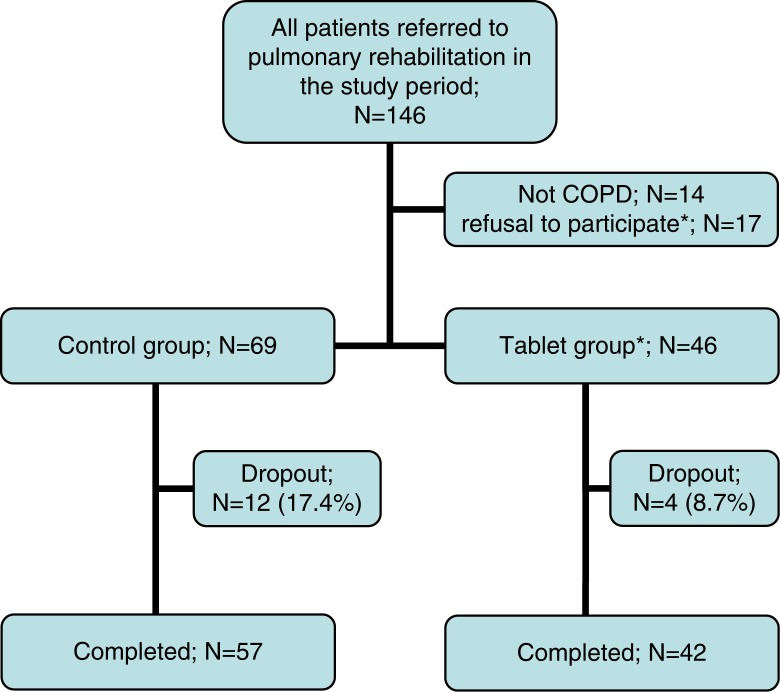
Flow chart of patients included in the 7-week pulmonary rehabilitation programme. *All from the tablet group.

A total of 115 patients with severe COPD were recruited from the pulmonary clinics of three hospitals in the Capital Region of Denmark (Hvidovre Hospital, Gentofte Hospital, and Nordsjælland Hospital) (Fig. 1). Eligibility criteria were stable COPD defined spirometrically as forced expiratory volume in 1 sec (FEV_1_) <80% of the predicted value and FEV_1_/forced vital capacity (FVC) <70%; motivation for PR. Exclusion criteria included muscoloskeletal, cardiac, and cognitive disorders that limited the ability of the patients to train and attend classes.

### Rehabilitation programme

Patients attended twice weekly for 7–10 weeks: 7 weeks at two hospitals (Gentofte Hospital and Hvidovre Hospital) and 10 weeks at Nordsjælland Hospital with each session lasting 2 h. Each session consisted of 1 h of supervised training and 1 h of education. The supervised training sessions consisted of walking and cycling. Patients were instructed to exercise at a level equal to 85% of predicted peak VO_2_ as calculated from the Incremental Shuttle Walk Test (ISWT). During the programme, total continuous walking times (with 85% of maximal intensity) were measured with a stopwatch and recorded in a diary both at the supervised training sessions and during unsupervised daily training at home (9, 10). The patient's diary was inspected at the supervised training sessions. Moreover, the patients received an illustrated folder with some of the exercises included in the supervised sessions. When the weather prevented them from walking outside, they were encouraged to use the stairs in their apartments or to do the exercises from the folder. Patients trained in groups of 10–14 individuals. During the programme, they were advised to increase the duration of the walk rather than the walking speed.

### Intervention

**Fig. 2:**
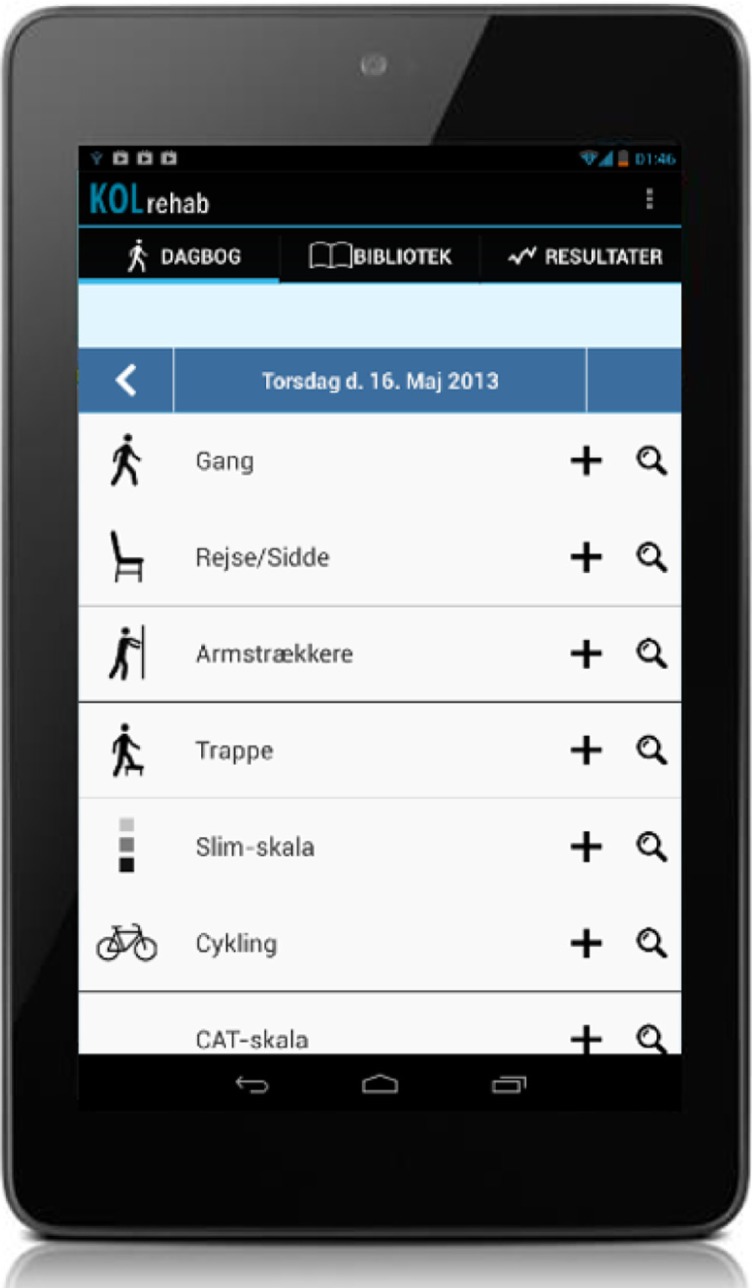
Nexus 7 tablet computer.

The study design was not randomised or blind. Every second training group was offered a tablet (minicomputer) – a 7-inch device (Asus Nexus 7) (Fig. 2). If patients in the intervention group did not want to participate, they were withdrawn from the study and were thus not considered as control (*N*=17). We aimed to have homogeneous training groups, that is, groups with all participants using or not using the tablets.

The tablet had a wireless, mobile-based Internet connectivity, which means that the patient is free to use it at home, in the hospital, or elsewhere.

The software consisted of an application that runs on a tablet computer (COPD_Rehab), as well as a web-based tool that also could be accessed by the rehabilitation staff (COPD_Rehab_Clinician) (see www.aidcube.com).

COPD_Rehab consisted of the following main components:

1. A training diary, training videos, and viewing training results. In the training diary, the patients could keep a record of their training activity, the types of exercises that are conducted including frequency and duration of the individual training sessions. In addition, the patients registered dyspnoea after each exercise using BORG dyspnoea score.
2. Video recordings of all the training exercises used. In this way, the patient had the opportunity to refresh how the exercise had to be performed, or he/she may choose to exercise while the video is playing.
3. Training results in graphical form, so that the patients could regularly monitor their own training.

COPD_Rehab_Clinician provided a possibility for the rehabilitation staff to monitor the patient's training. Weekly reports are sent to staff by the module, which provides an overview of how many activities an individual patient has done. In addition, the module provided access to view each patient's development in, for example, endurance shuttle walk time (ESWT). This knowledge was used to talk to the patients about their training and individual barriers and opportunities as well as to encourage further training. Staff praised participants with good results. Those with poor results or no results at all were contacted by the staff for clarification.

All the participants in the tablet group were taught basic use and the most important features in a session. The session changed between teaching and trying oneself with the possibility of instant help. In the following training session, the patients were asked to report their actual training using the device. In this way the participants were forced to use the device from the start. The use of the device facilitated the patients to help each other and gave a special sense of unity.

### Outcome measures

Primary effect parameters in the present trial were ESWT and disease-specific health status (COPD Assessment Test = CAT). ESWT and CAT were measured before and after rehabilitation. ISWT was used to measure the maximal exercise performance. The ESWT measured the sub-maximal exercise performance, when the patient walked at a constant speed equating 85% of the predicted VO_2_ peak as calculated from the ISWT.

The CAT is a disease-specific questionnaire, which comprises eight questions and has been demonstrated to be responsive in patients with COPD (11). The most reliable estimate of the minimum clinical important difference (MCID) of the CAT is 2 points (12).

Secondary effect parameters were dropout rates. The intervention group also reported the number of sessions and time spent on home exercise training. Satisfaction with the tablet was evaluated in 39 of 46 patients with a tablet from two hospitals.

### Sample size calculation

The overall power of the trial was determined on the basis of the primary outcome – ESWT. To detect a 60 s difference between the controls and tablet group on the ESWT at 7 weeks (standard deviation 85 s) at the 5% significance level with 90% power, 104 evaluable subjects were needed.

The locally appointed ethics committee has approved the research protocol, and informed consent has been obtained from the participating patients.

### Statistics

Data were analysed with the statistical package (SPSS) version 19.0 SPSS Inc., Chicago, USA. The chi-squared, two sample *t*-tests and Mann–Whitney U tests were used as appropriate to compare differences between groups, and Wilcoxon and one sample *t*-test were used to compare difference over time. A two-sided *p*-value of <0.05 was considered significant.

## Results

Table 1 shows the characteristics of the study population. Most of our patients had severe airflow limitation and dyspnoea while walking (93% had FEV_1_ ≤ 50% of predicted value, and 77% had a MRC score of 4–5) (Table 1).

**Table 1:** Patients’ characteristics at baseline

	Intervention, *n*=46	Controls, *n*=69	*p*-level for difference
Age, years (SD)	68.4 (9.1)	68.8 (11.0)	0.75
Gender, % females	44.6	60.9	0.07
FEV_1_ % predicted value	30.9 (9.1)	35.1 (10.5)	0.03
Body mass index, kg/m^2^	24.2 (5.3)	24.7 (6.2)	0.83
Current smokers, %	14.3	17.4	1.00
Long-term oxygen therapy, %	7.1	13.0	0.73
Medical Research Council (MRC) dyspnoea score, mean (minimum–maximum)	4.2 (2–5)	4.5 (2–5)	0.08
Incremental Shuttle Walk Test, meter (SD)	197.0 (102.8)	185.4 (120.3)	0.29
Endurance Shuttle Walk Test, seconds (SD)	175.5 (72.2)	176.4 (117.5)	0.35
CAT score (*n*=45; *n*=64)	19.7 (6.8)	18.9 (6.1)	0.54
Started rehabilitation between			
October 1 and March 1, %	21.7	44.9	0.01

Continuous variables are presented as mean (SD) unless otherwise indicated.

FEV_1_ % was lower in the intervention group and fewer patients started PR in the winter than in the control group, but these covariates were not associated with outcomes – dropout and changes in ESWT and CAT score.

Dropout rate was lower in the tablet group compared with the control group, but this difference did not achieve statistical significance (8.7% vs. 17.4%; *p=*0.19).

After PR, a statistically significant improvement was seen for ESWT 117.6 s (*p*<0.001) and for CAT 1.4 (*p*=0.02). In the control group, rehabilitation resulted in a significantly improved walking time (167 s (95% CI: from 103 to 231 s; *p*<0.001)) and no change in CAT score (−0.6 units (95% CI: from −2.3 to 1.0 units; *p*=0.43)). In the intervention group, the walking time increased by 51 s (95% CI: from 5 to 97 s; *p* = 0.03), and the CAT score decreased (improved) by 2.3 units (95% CI: from 0.6 to 3.9 units; *p*<0.01). The change in the walking time was significantly better in the control group compared with the intervention group (*p*<0.01), whereas the change in CAT score did not differ significantly between the two groups (*p*=0.17).

**Fig. 3:**
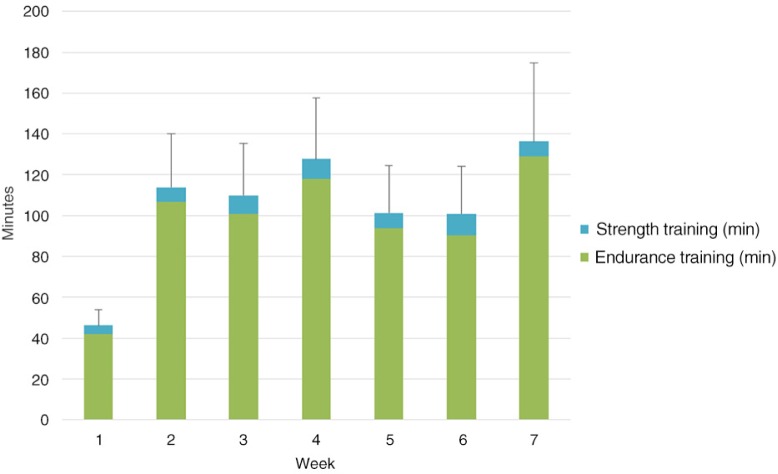
Changes in self-reported time with unsupervised endurance and strength training at home over time in patients with a tablet (columns are mean values and sticks are standard errors).

Figure 3 shows the reported duration of the endurance and strength training during the first 7 weeks for the 40 patients who completed the rehabilitation programme (two patients attended the evaluation visit but were not able to perform the ESWT). The first week patients reported significantly less time with training (in average 46.2 min) compared with the other weeks (101.0–136.2 min).

The questionnaire focusing on the experience and satisfaction with the tablet computer was filled in by 33 of 36 patients who completed the study. Before the enrolment in the rehabilitation programme, eight (24%) already had a tablet, two (6%) had a smartphone, and 19 (58%) had a personal computer.

**Fig. 4:**
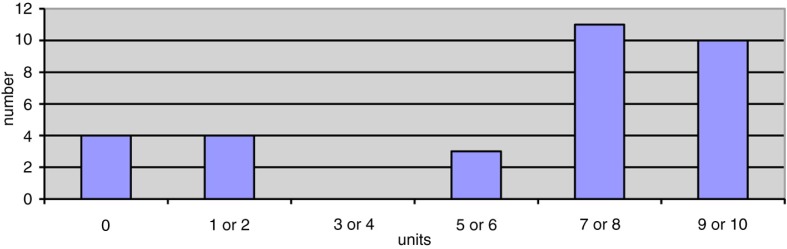
Patients’ satisfaction with the table according to a visual analog scale from 0 (very difficult to use) to 10 (very easy to use), *N*=32 patients.

**Fig. 5:**
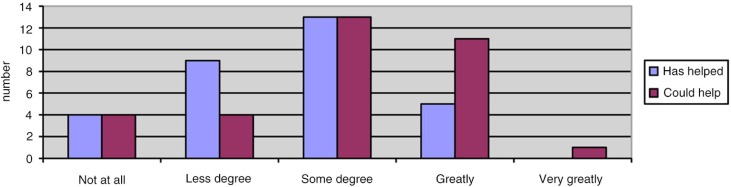
Answers to whether the tablet has helped (*N*=31) and could help (*N*=33) the patient to remain physically active in the future.

Figure 4 shows patients’ satisfaction with the tablet. Eighteen (56%) patients reported a score ≥8, where 10 denoted ‘extremely satisfied’. Figure 5 shows patients’ answer as to whether the tablet could help them to remain physically active in the future. Eight (24%) patients answered ‘not at all’ or ‘to a lesser degree’.

## Discussion

We found that a supplementation of PR with a tablet with instructions and a training diary did not improve the walking distance or the health-related quality of life more than a usual 7-week PR programme without a tablet. Actually, control patients gained significantly better walking time compared with patients with the tablet. Compared with controls, there was a tendency that more patients in the tablet group attended the evaluation visit. A part of the explanation may be that these patients had to return the tablet after the study period. We have previously shown that patients with exacerbation of COPD and poor yield of rehabilitation frequently absent from the evaluation visit. Therefore, the proportion of patients who turn up for the evaluation visit will affect the changes in the ESWT and CAT (10). Thus, we expect that the higher attendance in the tablet group will result in a poorer average response to PR in this group.

We found a significant improvement in CAT score, and in the tablet group it was above MCID (2 points) and similar to findings from a large multicentre study where CAT score was improved by 2.6 points after 8 weeks of rehabilitation (13).

Most rehabilitation programmes have either supervised exercise training combined with self-monitored training at home (14–16) or entirely self-monitored home-based exercise training (17). Adherence to supervised training is often high (14, 18). However, the literature on adherence to self-monitored training at home is sparse. Tabak et al. evaluated a telehealth programme including an activity coach for ambulant activity monitoring, real-time coaching of daily activity behaviour, and web-based exercise programme for home exercising in 29 COPD patients (19). The programme was assessed most of the days, but adherence to prescribed exercise scheme was low (21%). In contrast, a study from Taiwan found that all 48 COPD patients were adherent to a daily cell phone-based exercise programme for 3 months (20), and a pilot study from Canada found that adherence rate to unsupervised exercise training was 80% (18).

Our patients reported via the tablet that they exercised on average 1.5–2 h per week at home. One of the main aims of the tablet was to motivate patients to increase home training via various exercises and ongoing reporting of home training to the rehabilitation staff. Therefore, we were disappointed to see that the reported exercise time in the tablet group only increased significantly after the first week and then remained constant. Other studies have shown equivocal results of web-based exercise programme for home exercising (19, 20). Activity level (steps per day) was not significantly affected by intervention in the previously mentioned study by Tabak (19). However, an Internet-mediated pedometer-based programme improved daily step counts after 4 months in a large study of 239 COPD patients (21).

We have no data on how the staff perceived the intervention. In general, participants’ satisfaction was high, as shown in previous studies (8, 18, 19, 22). However, in line with the study by Paneroni et al., we found that about one quarter of the patients were disappointed with the tablet and stated that it would not help them (22). However, it is necessary to be innovative and think differently if the real potential of implementing a device like this in rehabilitation is to be achieved. It takes time and experience to use the new possibilities both regarding planning education and empowering patients.

Selection of patients may account for different results of telehealth care. Among the previous studies, only Tabak et al. and Nguyen et al. have reported the number of patients who were not able or not willing to participate, and in these studies only 29 and 40% of the referred patients participated (7, 19). In fact, Nguyen et al. included a run-in period of 2 weeks to make sure that the patients were able and willing to continue the telemonitoring (7).

A potential limitation of our and of the two mentioned studies (19, 20) is the fact that we relied on self-reported home training. Another limitation is the study design, which was controlled but not randomised nor blinded. Before rehabilitation, refusal to participate was only seen in the tablet group and thus a clear patient selection with inequalities for gender, FEV1, and winter rehabilitation. Finally, we have only studied the effect during 7–10 weeks of rehabilitation – a relatively short period. One should consider that an application to facilitate home exercise may be more effective compared with usual care when the supervised part of a rehabilitation programme ends, and it may take some time before patients learn how to utilise the facilities of the tablet. Several of our patients reported that they expected the tablet to help them to stay physically active in the future.

Since this application was developed 3 years ago, the application has gone through an extensive development based on feedback from patients and healthcare professionals. Therefore applications and electronic devices have generally increased their usefulness. This specific application represents one of the many possibilities for supporting home-based PR. It is probably most useful for patients that already possess a certain level of IT skills, whereas other types of technology such as live video exercise sessions might be more suitable for patients with a lower level of IT skills. It would also be interesting to incorporate the use of activity trackers in this kind of application (Fitbit, Jawbone, etc.), which will provide objective exercise tracking and a ‘physically visible’ reminder and possibly be an extra motivation for some patients.

This is the first study to evaluate the effect of adding a tablet with instructions and training diary to a classic 7–10 weeks outpatient PR programme and can therefore be regarded as a pilot study. The next step would be to include patients motivated to use a tablet with the latest applications in a randomised study with exercise tolerance, physical activity, and health quality of life as outcomes. In conclusion, compared with the usual PR, no significant improvements were seen in the group equipped with the tablet after 7–10 weeks of rehabilitation. Future studies should focus on long-term effects.
